# Transcultural Adaptation and Theoretical Models of Validation of the Spanish Version of the Self-Care of Heart Failure Index Version 6.2 (SCHFI v.6.2)

**DOI:** 10.3390/ijerph18020569

**Published:** 2021-01-12

**Authors:** Raúl Juárez-Vela, Angela Durante, Rosa Antonio-Oriola, Vicente Gea-Caballero, Michał Czapla, Iván Santolalla-Arnedo, Regina Ruiz de Viñaspre-Hernández, Amaya Burgos-Esteban, José Vicente Benavet-Cervera, Jorge Rubio-Gracia, Ercole Vellone

**Affiliations:** 1 Department of Nursing, University of La Rioja, 26006 Logroño, La Rioja, Spain; raul.juarez@unirioja.es (R.J.-V.); ivan.santolalla@unirioja.es (I.S.-A.); reruizde@unirioja.es (R.R.d.V.-H.); amaya.burgos@unirioja.es (A.B.-E.); 2 Group of Research in Sustainability of the Health System, Center of Biomedial Research of La Rioja—CIBIR, 26006 Logroño, Spain; 3 Department of Biomedicine and Prevention, University of Rome Tor Vergata, 00133 Rome, Italy; angela.durante@uniroma2.it (A.D.); ercole.vellone@uniroma2.it (E.V.); 4 Hospital Lluís Alcanys, Xativa, 46800 Valencia, Spain; 5 Nursing School La Fe, Adscript Center Universidad de Valencia, 46026 Valencia, Spain; 6 Research Group GREIACC, Health Research Institute La Fe, 46026 Valencia, Spain; 7 Faculty of Health Sciences, Wroclaw Medical University, 50416 Wroclaw, Poland; michal.czapla@umed.wroc.pl; 8 Department of Health Sciences, VIU Valencia International University, 46003 Valencia, Spain; josevicente.benavent@campusviu.es; 9 Internal Medicine Service, Hospital Clinico Lozano Blesa, 50009 Zaragoza, Spain; jorgerubiogracia@gmail.com; 10 Heart Failure Group, Research Institute of Aragon IIS Aragon; 50009 Zaragoza, Spain

**Keywords:** self-care, heart failure, psychometrics

## Abstract

Background: Heart failure (HF) is a major and growing public health problem worldwide. Across the world, heart failure is associated with high mortality, high hospitalization rates, and poor quality of life. Self-care is defined as a naturalistic decision-making process involving the choice of behaviors that maintain physiologic stability, the response to symptoms when they occur, and the ability to follow the treatment regimen and control symptoms. One instrument used to measure self-care is the Self Care of Heart Failure Index. Aim: The purpose of this study was to test the psychometric properties of the Spanish version of the Self Care of Heart Failure Index v.6.2 (SCHFI v.6.2). Methodology: Before testing its psychometric properties, the SCHFI v.6.2 was translated and adapted from its original English version into Spanish. Subsequently, we tested the instrument’s psychometric properties on a sample of 203 participants with HF. Descriptive statistics were used to analyze the sociodemographic and clinical variables, and to describe item responses. We tested the factorial validity of the SCHFI v.6.2 using confirmatory and exploratory factor analysis. Results: Confirmatory factor analysis (CFA) was performed using the our pre-existing models which resulted with poor fit indices. Thus, we performed exploratory factor analysis (EFA) on each of the SCHFI v.6.2 scales. Conclusion: The Spanish version of the SCHFI v.6.2. has good characteristics of factorial validity and can be used in clinical practice and research to measure self-care in patients with HF.

## 1. Introduction

Heart failure (HF) is a major and growing public health problem worldwide. In developed countries such as the United States, HF affects approximately 2% of the adult population [1], rising to about 10% in people age 70 or older. It is estimated that approximately 15 million Europeans suffer from HF, with a prevalence that ranges from 0.4% to 2.3% in European populations [2]. In Spain, the prevalence of HF is 1.3% in people between 45 and 54 years but rises to 16.1% in people over the age of 75 [3]. Across the world, HF is associated with high mortality, high hospitalization rates, and poor quality of life [4]. In Spain, HF is the first cause of hospitalization in adults over age 65 and represents 2% of the total healthcare budget [2].

Self-care has been shown to improve quality of life and reduce hospitalization rates in patients with HF [5,6]. Defined as a naturalistic decision-making process, self-care involves the choice of behaviors that maintain physiological stability (self-care maintenance) and the response to symptoms when they occur (self-care management) [7]. Both self-care maintenance and self-care management are influenced by self-care confidence which is the self-efficacy in performing self-care [7,8]. Naturalistic decision making describes how people make decisions in real-world settings [9].

One instrument used to measure self-care is the Self Care of Heart Failure Index (SCHFI). This instrument, developed in the USA, was initially comprised of 15 items divided into three scales. This version of the SCHFI was tested in a U.S. sample of 760 HF patients [10], and was updated to version 6.2, in 2009. Version 6.2 has 22 items divided into the following three scales: self-care maintenance, self-care management, and self-care confidence. The total score for each separate scale is standardized from zero to 100 [11]. Since then, the instrument has been translated into several other languages [12,13,14,15].

Spanish is the second most common spoken language in the world [16], but the psychometric properties of the Spanish version of the SCHFI have, until now, not been described.

Self-care behaviors are influenced by language and culture [17]. For this reason, it is important to translate, culturally adapt, and assess the psychometric properties of the SCHFI across other countries and languages. Adapting an instrument from one language to another is a common practice, but after doing so, investigators must retest psychometric characteristics to assure equivalence [18]. Therefore, the purpose of this study was to test the psychometric properties of the Spanish version of the SCHFI v.6.2.

## 2. Methods

### 2.1. Translation, Adaptation, and Modeling

Before testing its psychometric properties, the SCHFI v.6.2 was translated and adapted from its original English version into Spanish. We followed the guidelines published by Beaton et al. [19], which divided the process into the following six steps, namely: (1) translation, (2) synthesis, (3) back translation, (4) synthesis of back translation, (5) expert committee review of the translated version, and (6) pretesting.

According to this methodology, the original SCHFI v.6.2 was translated into Spanish by a researcher who was familiar with the instrument and its characteristics. This Spanish translation was blindly back translated into English by a bilingual researcher who had not seen the original English version. Both researchers were instructed to use simple sentences, and avoid metaphors, colloquial terminology, passive sentences, and hypothetical statements. The back-translated version of the SCHFI was reviewed by the original author of the instrument to check the accuracy of the translation. Minor translation issues were resolved by e-mail and a final Spanish version of the SCHFI v.6.2 was established. Subsequently, an expert committee compared and contrasted both the original and back-translated versions of the SCHFI and agreed, by consensus, on a final Spanish version of the SCHFI v.6.2. The objective of the expert committee was the adaptation as precisely as possible to the original language of the Spanish version of the SCHFI. It was made up of native teachers in both languages with clinical experience. As a final stage, cognitive interviews were completed on a sample of 32 patients. In this phase, minor changes were made to the translation in order to improve the readability of the items. For example, to clarify the differences between the items measuring “exercise” and “physical activity”, we added some examples of physical activity (i.e., gardening and housekeeping).

### 2.2. Procedures and Statistical Analysis

This study was conducted in the northeast region of Aragon (Spain) using a cross-sectional design.

We enrolled a sample of *n* = 203 participants admitted to the Hospital Clínico Lozano Blesa in Zaragoza (Spain), who met the following inclusion criteria: (1) being diagnosed with HF according to the European Society of Cardiology (ESC) criteria [2], and (2) being 18 years or older. We excluded patients with a significant cognitive impairment established by scoring less than 4 points on the six-item screener [20]. All data were collected by qualified nurses, who had been specifically trained for this purpose, during the patients’ admission. Once the patients had granted their informed consent for participation in the study, they were interviewed. The study was conducted during 2018.

All participants completed the Spanish version of the SCHFI v.6.2, comprising its three scales, i.e., the self-care maintenance scale (10 items), the self-care management scale (six items), and the self-care confidence scale (six items). Each item uses a five-point Likert scale for responses. We also administered a sociodemographic questionnaire to collect characteristics and factors related to HF such as age, smoking habit, number of previous hospitalizations, marital status, and level of education.

Sociodemographic and clinical variables were summarized using descriptive statistics such as mean and standard deviation in the case of quantitative variables, and frequencies in the case of categorical variables. In addition, descriptive statistics were used to describe item responses and to summarize scale scores.

We tested the factorial validity of the SCHFI using factor analysis. Initially, we tested the previously published SCHFI models [21,22] using confirmatory factor analysis (CFA). However, the fit for these models was poor for the Spanish version of the SCHFI. Subsequently, we performed exploratory factor analysis (EFA) in order to determine the number of latent constructs and the underlying factor structure of each SCHFI v.6.2 scale. For the CFA, we used the following fit indices: (1) χ^2^ test, non-significant values are interpreted as supporting model fit; (2) comparative fit index (CFI), values ≥ 0.90 or >0.95 support good fit; (3) normed fit index (NFI), values ≥ 0.90 support good fit; and (4) root mean square error of approximation (RMSEA), values < 0.06 indicate good approximation of fit. For the EFA, we used principal axis factoring and ProMax oblique rotation. Data analysis was performed using SPSS and IBM SPP-AMOS V24 (IBM Corporation, New Orchard Road Armonk, New York, NY, USA).

### 2.3. Ethical Considerations

This study adhered to European and Spanish data protection regulations (Organic Law 3/2018 and General Data Protection Regulation (EU) 2016/679). The study protocol was reviewed and approved by a local research ethics committee (reference no. P15/0216). A local ethics committee approved the study before data collection began. All participants were fully informed about the aims of the study and signed the informed consent form prior to completing the research instruments. Participation was voluntary, and confidentiality and anonymity were safeguarded at all times.

## 3. Results

Table 1 illustrates the main sociodemographic characteristics of the sample. There were slightly more men than women (50.2%) and the mean age of the sample was 81.10 years. Most of the subjects were widowers (46.8%) and they were mostly educated up to a primary school level (87.2%).

The mean, standard deviation (SD), skewness, and kurtosis values for the Spanish version of the SCHFI are reported in Table 2. Regarding the self-care maintenance scale, the item with the highest score was “keep doctor or nurse appointments”, whereas the item with the lowest score was “exercise for 30 min”. In the self-care management scale, the item “call the physician or nurse” in the case of symptoms had the highest score, whereas item Number 16, i.e., evaluating symptom treatment, had the lowest score. Finally, in the self-care confidence scale, the item with the highest score was Number 18, evaluating confidence in following the treatment advice, and the item with the lowest score was Number 21, evaluating how well a remedy works.

The CFA was performed initially on the previously published SCHFI models [12,21,22] model fit was determined by combining information from the following exact fit statistics: Chi-square test (χ^2^), comparative fix index (CFI), normed fix index (NFI), and root mean square error of approximation (RMSEA). However, the fit indices of the above models were all poor (Table 3).

Model 1 was tested with the three SCHFI v.6.2 scales in a single model as in Riegel et al.; Model 2 was tested performing separate confirmatory factor analyses, one per each scale and in Vellone et al.; Model 3 was tested with 2 factors per each SCHFI v.6.2 scale according to Vellone et al.; Model 4 was tested with four factors in self-care maintenance scale, two factors in self-care management scale and one factor in self-care confidence scale as in Barbaranelli et al. Self-care management model in Model 3 and 4 was not identified.

Since none of the tested models obtained supportive fit indices, we performed exploratory factor analysis (EFA) on each of the SCHFI v.6.2 scales (see Table 4, Table 5 and Table 6). In deciding the best factor solution of the EFA, we considered the following criteria: (1) factor loading >0.30, (2) the number of items per factor, (3) the interpretability of the solution, (4) the scree plot of the eigenvalue, and (5) the theory underpinning the SCHFI. According to these criteria, the best solution was identifying two factors for each of the SCHFI v.6.2 scales. In the self-care maintenance scale, the first factor included Items 1,2 3, 5, 6, and 10. This factor was named “illness behaviors”. The second factor included Items 4, 7, and 9, and was named “health promotion behaviors”. In the self-care management scale, we identified two factors. The “prevention behaviors” factor included Items 11,12, and 15, and the “illness behaviors” factor included Items 13, 14, and 16. Finally, two factors were also identified in the self-care confidence scale, namely the factor called “targeted prevention behaviors”, which included Items 17, 18, 19, and 20, and the factor called “autonomous prevention behaviors”, which included Items 21 and 22.

## 4. Discussion

The objective of this study was to carry out the cross-cultural adaptation and validation into Spanish of the SCHFI v.6.2, and to improve our understanding of the dimensions measured by the SCHFI v.6.2 (self-care maintenance, self-care management, and self-care confidence), thus, obtaining a culturally equivalent instrument to assess self-care skills, which implies the choice of behaviors that maintain physiological stability, the response to symptoms when they occur, and the ability to follow the treatment regimen and control symptoms to avoid HF decompensation. To our knowledge, this is the first study validating the SCHFI v.6.2 in Spanish language. To date, similar validation studies, outside the U.S, have been conducted only in Italy [12], Brazil [13], Iran [14], and China [15].

During the cross-cultural adaptation process, some terms and expressions were modified to ensure cultural equivalence to clarify the differences between the items that measured “exercise” and “physical activity”. To improve understandability in our environment, these adjustments were addressed by adding some examples. Once translated and back translated, items were evaluated in a representative sample of patients residing in the northeast region of Aragon (Spain).

Regarding the characteristics of the participants, in our study, there were more men than women, with a mean age of 81.10 years, a low educational level, and, as expected, in a situation of work inactivity. This typology of patients differs greatly from that of the sample used by Riegel et al. in the original construction and validation of the questionnaire (they were younger, and most had secondary education). The sample was by far the oldest as compared with the other validations, including the original, from 55.8 years in the Chinese sample to 72.73 years in the Italian sample. Regarding the distribution by sex, the sample presented similar characteristics to that of the original study. The great predominance of men over women in the sample of Brazil (78.9%) and China (71%) stands out.

In the American sample, the educational level was higher; the majority had secondary and higher education, which represented a clear difference for the study. In the validations carried out in Italy and China, there was a great predominance of patients with primary school level studies (more than 74%). This difference can be explained, in particular, in the Spanish sample by taking into account the age differences.

The initial CFA test of the three SCHFI scales in a single model to test factor validity resulted in a poor fit for the Spanish version, as did the tests of the other existing models. This was not completely unexpected because self-care is influence by several factors including culture, patient education, and the health care systems [7].

The self-care maintenance scale revealed two factors that we called disease behaviors and health-promoting behaviors. Items related to “exercise and activity” were separated from the other items. It should be taken into account that, in Spain, exercise is seen as a benefit by the younger groups but it is not a lifestyle of the elderly, therefore, the result may be due to the lack of availability of cardiac rehabilitation programs, as in the Chinese study, but it may also be because the main symptom of chronic HF is the limited ability to exercise. An item that had an unexpected result was “Ask for low-salt items when eating out or visiting others”. This may be related to the work situation of the sample since most were in a situation of inactivity, which implies family meals and few events.

The factor analysis of the self-care management scale revealed two factors that we called the “prevention behaviors” factor and the “illness behaviors” factor. It was unlikely that the patients would reduce their “fluid intake and/or increase the dose of diuretics”, as well as “evaluate the effectiveness of the treatment”. This could be due to the fact that self-care management recommendations are not a common practice in Spain and in Italy, as described by Da Conceicao et al. [23] and Cocchieri et al. [24]. This was also reflected in the European study and the Brazilian study in relation to the item “take an additional diuretic”, which reflects differences in the treatment norms that may be related to the country’s health system.

The factor analysis of the self-care confidence scale revealed two factors that we called “targeted prevention behaviors” and “autonomous prevention behaviors”, which again did not differ from the results of the Italian study, separating two items that require training, i.e., “do something to relieve your symptoms” and “evaluate how well a remedy works”, from the rest of the items that did not require making decisions.

Confirmatory factor analysis in the Brazilian, Persian, and Chinese versions revealed that the SCHFI v.6.2 models they tested was quite similar to the original testing, but this was not the result of our study, since the previously published SCHFI models [21,22] did not achieve the expected results with supportive fit indices. When the EFA was performed on each of the SCHFI v.6.2 scales, a different factorial structure emerged which was different form the published models.

## 5. Limitations

The study has some limitations that are important to highlight. The sample size, although sufficient to assess the main objectives of the study, could be improved by the addition of more participants. However, in the literature, only Italy has a bigger sample size. The studies of Brazil, China, and Iran had smaller samples.

We encountered some difficulties in applying the instruments. The low educational level of most of the respondents forced us to have a proactive attitude, i.e., offering help, but having an objective attitude in their administration. Through this, we were able to induce certain answers, although we tried to maintain an objective attitude at all times and only intervened when the respondent requested it and in order to clarify the meaning of any of the questions, rather than to induce or favor certain answers. It would be beneficial to carry out more studies in different regions.

## 6. Conclusions

As compared with the rest of the validations, the results of transcultural validation were better than the Persian, Chinese, and Brazilian versions and were similar to the Italian version. Our study has shown that the Spanish version of the SCHFI v.6.2. has a factorial validity and could be used in clinical practice and research to measure self-care in patients with HF.

## Figures and Tables

**Table 1:** Main sociodemographic characteristics of the sample (*n* = 203).

**Variables**	***n***		**%**	
Female	101		49.8	
Male	102		50.2	
Single	15		7.4	
Married	92		45.3	
Divorce	1		0.5	
Windover	95		46.8	
**Education Level**				
Primary school	177		8.2	
Secondary school	13		6.4	
Vocational education and training	1		0.5	
General certificate of education	6		3	
University	6		3	
**Work**				
Employed worker	3		1.5	
Self-employed	5		2.5	
Pensioner	193		95.1	
Unemployment	2		1	
**Do You Smoke Currently?**				
Yes	13		6.4	
Not	188		92.6	
**Do You Drink Alcoholic Drinks?**				
Yes	12		5.9	
Not	189		93.1	
	**AGE**	**HEIGHT**	**WEIGHT**	**How many DAYS have you been hospitalized during last 12 months due to heart failure (HF)?**
	**(years)**	**(cm)**	**(kg)**	
25th Percentile	76.00	150.00	67.00	7.00
50th Percentile	83.00	160.00	76.00	10.00
75th Percentile	87.00	169.00	87.30	14.75

**Table 2:** Descriptive statistics of the Self Care of Heart Failure Index (SCHFI) items.

	Mean	SD	Skewness	Kurtosis
Self-care maintenance scale				
Listed below are common instructions given to persons with heart failure. How routinely do you do the following?				
1. Weigh yourself	2.14	1.389	0.759	−0.860
2. Check your ankles for swelling	3.2834	1.49547	−0.388	−1.335
3. Try avoid getting sick	3.3155	1.72643	−0.321	−1.654
4. Do some physical activity	2.4225	1.33929	0.453	−0.954
5. Keep doctor or nurse appointments	4.3155	1.28345	−1.843	1.987
6. Eat a low-salt diet	2.9198	1.58249	0.084	−1.572
7. Exercise for 30 min	1.6952	1.26071	1.732	1.622
8. Forget to take one of your medicines	1.8182	1.42900	1.453	0.469
9. Ask for low-salt items when eating out or visiting others	2.0267	1.37345	0.983	−0.447
10. Use a system (pill box…) to help you remember your medicines	3.6738	1.68022	−0.739	−1.183
Self-care management scale				
In the past month have you had trouble breathing or ankle swelling? ^†^				
11. If you had trouble breathing or ankle swelling in the past month, how quickly did you recognize it as symptoms of HF?	2.5357	1.95731	0.011	−1.599
Listed below are remedies that people with HF use. If you have trouble breathing or ankle swelling, how likely are you to try one of these remedies?				
12. Reduce the salt in your diet	2.6150	1.70432	0.381	−1.600
13. Reduce your fluid intake	1.6471	1.24587	1.843	2.010
14. Take an extra water pill	1.5294	1.14203	2.138	3.295
15. Call the physician or nurse	4.1872	1.47095	−1.486	0.485
Think of a remedy you tried the last time you had trouble breathing or ankle swelling.				
16. How sure were you that the remedy helped or did not help?	1.4064	1.47581	0.834	−0.330
Self-care confidence scale				
In general, how confident are you that you can…				
17. Keep yourself free of heart failure symptoms	2.8021	1.33537	−0.002	−1.316
18. Follow the treatment advice you have been given	3.4332	1.41405	−0.595	−0.930
19. Evaluate the importance of your symptoms	2.8503	1.26535	−0.165	−1.158
20. Recognize changes in your health if they occur	2.8396	1.30998	−0.163	−1.255
21. Do something that will relieve your symptoms	2.0535	1.23014	0.896	−0.354
22. Evaluate how well a remedy works	1.7647	1.17694	1.408	0.885

† This question is used to identify patients who reported symptoms. The self-care management scale can be completed only if patients reported symptoms during the last month.

**Table 3:** CFA fit indices of the tested SCHFI v.6.2 models.

Models	χ^2^ (*p* Value)	DF	CFI	NFI	RMSEA
Model 1	785.842 (<0.001)	206	0.627	0.560	0.123
Model 2					
Self-care maintenance scale	189.160 (<0.001)	35	0.573	0.545	0.148
Self-care management scale	52.949 (<0.001)	9	0.624	0.616	0.155
Self-care confidence scale	137.585 (<0.001)	9	0.816	0.809	0.266
Model 3					
Self-care maintenance scale	95.629 (<0.001)	32	0.828	0.770	0.103
Self-care management scale					
Self-care confidence scale	137.425 (<0.001)	8	0.817	0.809	0.295
Model 4					
Self-care maintenance scale	75.041 (<0.001)	21	0.853	0.814	0.118
Self-care management scale					
Self-care confidence scale	137.527 (<0.001)	9	0.817	0.809	0.277

Note. χ^2^ = chi square test; DF = Degree of Freedom; CFI = Comparative Fit Index; NFI = normed fit index; RMSEA = Root Mean Square Error of Approximation.

**Table 4:** Exploratory factor analysis of the self-care maintenance scale.

Items	Factor Loadings (Standardized Betas)	
	Factor 1	Factor 2
1	**0.353**	0.213
2	**0.532**	0.162
3	**0.511**	−0.058
4	0.056	**0.785**
5	**0.768**	0.007
6	**0.578**	0.191
7	−0.109	**0.680**
8	0.003	0.142
9	0.090	**0.450**
10	**0.691**	−0.291

Factor 1, illness behaviors and Factor 2, health promotion behaviors.

**Table 5:** Exploratory factor analysis of the self-care management scale.

Items	Factor Loadings (Standardized Betas)	
	Factor 1	Factor 2
11	**0.522**	−0.150
12	**0.880**	0.238
13	−0.001	**0.659**
14	−0.309	**0.703**
15	**0.407**	−0.124
16	0.117	**0.263**

Factor 1, prevention behaviors and Factor 2, illness behaviors.

**Table 6:** The exploratory factor analysis of the self-care confidence.

Items	Factor Loadings (Standardized Betas)	
	Factor 1	Factor 2
17	**0.844**	−0.066
18	**0.743**	−0.014
19	**0.939**	−0.018
20	**0.817**	−0.111
21	0.163	**0.766**
22	−0.123	**0.951**

Factor 1, targeted prevention behaviors and Factor 2, autonomous prevention behaviors.

## Data Availability

The anonymous data presented in this study is available upon request from the first author. The data is not publicly available due to the legislation on personal data protection and current legislation.
